# Fluid flow lifting a body from a solid surface

**DOI:** 10.1098/rspa.2018.0286

**Published:** 2018-11-21

**Authors:** Samire Balta, Frank T. Smith

**Affiliations:** Department of Mathematics, University College London, London WC1E 6BT, UK

**Keywords:** fluid, body, interaction, granular media, analysis

## Abstract

If a body is at rest on horizontal ground and a sudden horizontal flow of fluid is applied, the body either remains on the ground (rocking, rolling, sliding or spinning) or is lifted off impulsively. This lift-off is followed by a return to the ground or by a fly-away in the sense of continued departure from the ground. Related phenomena arise in the lift-off of an air vehicle from, effectively, moving ground. The present investigation seeks fairly precise mechanistic conditions under which lift-off and subsequent return or fly-away occur for a thin body or more generally for any thin gap of fluid between a body and the ground. Nonlinear fluid–solid interaction takes place in which the motion of the body and the surrounding fluid affect each other. Small-time analysis on lift-off and a numerical study are presented, followed by large-time analysis showing a critical flow speed for fly-away for any shape of the body. The changes in ground effect, from being dominant during lift-off to diminishing in fly-away, are explored together with relevant applications.

## 1. Introduction

The interest here is in an impulsive fluid flow removing a body originally stationary on a fixed solid surface. The body is supposed to be much denser than the fluid, such that gravity can affect the body movement appreciably whereas the fluid flow feels almost no gravity effect over the current time frame. The mechanisms for a single body in two-dimensional flow are studied by modelling, analysis and related computation, with a view to understanding lift-off followed by either a return to the surface (ground) or complete fly-away of the body. Experimental results including saltation, take-off, entrainment are quite plentiful as in [1–6] and further pioneering experiments and related work are in [7–9]. Of special interest are the studies of body shape effects experimentally and numerically in [7], of turbulent-flow effects on the threshold of motion in [8] and of sheared-flow effects for spherical particles in [9]. This last interesting recent paper (also see [10,11]) on experiments and related modelling pointed out that, in quantitative terms, the conditions required for fluid-driven removal of a particle from a solid surface were not well established and that there existed then no analytical results for configurations where fluid inertia is important (as is the case here).

The approach taken in the present paper is an alternative approach which is based on describing quantitatively the physical response of the thin layer of fluid supporting a body as lift-off occurs, our description being by means of model analysis supported by reduced computations and certain experimental links. This follows the fascinating results and motivation above and is intended to be complementary to the previous studies. A significant aspect of the present investigation is the ground effect coupled with the sudden horizontal flow. The present contribution is also associated with the model in [12], a paper which mentions many other applications including the relevance to the movement of dust on the planet Mars. The work in [12] is for unsteady interactions prior to lift-off: however, after lift-off, the fluid gap opens up and so there is no contact point in the present setting.

This paper considers phenomena that are dominated by unsteady, momentum and pressure forces. Applications arise in the removal of debris, grain segregation, dust blowing, leaf-blowers, sand movement, ski jumping or aircraft take-off. See for example [13–15].

**Figure 1.**
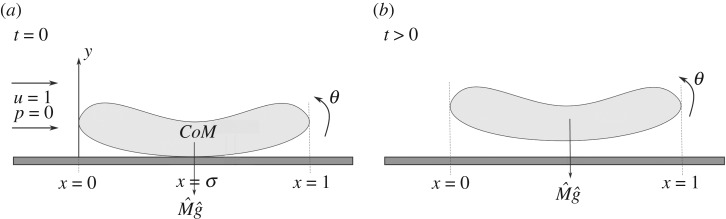
(*a*) A sketch of the thin body at its initial position, the fixed centre of mass (*CoM*), the contact point *x* = *σ* at time *t* = 0 and the oncoming stream of fluid. (*b*) The body position at some time *t* > 0.

The fluid is taken to be incompressible and Newtonian with uniform density (area density) ρ*, where the asterisk (*) refers to a dimensional quantity. The motion of the fluid and the immersed thin body (see figure 1) is expressed in terms of non-dimensional flow velocities (*u*, *v*), corresponding Cartesian coordinates (horizontal *x*, vertical *y*), time *t* and pressure *p*, such that the dimensional versions are u*(*u*, *v*), l*(*x*, *y*), l*t/u* and ρ*u*^2^*p*, respectively. Here u* is the free-stream fluid velocity, while l* is the length of the body and the temporal factor l*/u* is the typical transport time. In particular (*u*, *v*) is given by (1, 0) in the far field and the leading edge of the solid object can be taken as the origin. The Reynolds number *Re* = u*l*/ν*, where ν* is the kinematic viscosity of the fluid, is assumed large, in line with experiments. As a first model or approximation, an inviscid separation-free theory is applied, given that in many situations of real concern wall layers are turbulent and less prone to separate [16–18] than are laminar layers. A subsequent model for the laminar regime using ideas similar to those employed here would be called upon to cope with local flow separation perhaps by means of free-streamline theory.

Our interactions are governed by a nonlinear evolutionary system for the unknown scaled functions *h*, *θ*, *u*, *p*. Here *h*(*t*) is the vertical *y*-location of the centre of mass of the body, while *θ*(*t*) is the small angle the body chord makes with the horizontal. Also, *x* = *σ* is the prescribed *x*-location of the centre of mass and the initial contact point with the ground. Similar interactions arise in [19–21] in various different contexts of fluid–solid interplay.

Section 2 presents the model in detail including the description of the fluid–body interaction. The reasoning here is mostly expressed in terms of a thin body nearly aligned with the uniform incoming fluid flow but similar considerations apply for a thicker body provided that the gap between the under surface of the body and the ground is relatively small. This is followed by §3 which studies the behaviour for small times. Section 4 examines the lift-off criterion for different body shapes by means of the general formula derived which is then applied to specific examples. If the body does lift off, it returns to the ground within a finite time or flies away at large time (§§5 and 6). We focus on the latter, finding a criterion for fly-away. The final §7 provides the conclusions including discussion of flow separation and other features concerning the physical validity of the lift-off and fly-away criteria.

## 2. The fluid–body interaction

The body is assumed to have a smooth shape with a non-dimensional horizontal length of unity and is thin, of vertical scale *O*(*δ*) for *δ*≪1. The incoming flow moving from left to right is the uniform stream with (*u*, *v*) = (1, 0). Thus, the incoming vorticity of the flow is predominantly zero, with the majority of the thin body being assumed to be located in a region outside any oncoming turbulent or laminar boundary layer beneath the oncoming free stream, or possibly inside the outer portion of a turbulent boundary layer where the velocity deficit from the free-stream value is small. The present setting contrasts with that in the recent work [22,23] where non-zero incoming vorticity due to a boundary layer or channel flow is included. In the present setting, the body is initially in contact with the fixed horizontal surface *y* = 0 at its centre of mass whose *x*-location is *x* = *σ* as shown in figure 1.

Concerning figure 1, the scaled body mass is represented as $\hat{M}$ and moment of inertia as $\hat{I}$ (see details below) while $\hat{g}$ denotes the scaled acceleration due to gravity. We should remark that in effect the mass and other quantities involved are non-dimensionalized first and then if necessary (to take account of any small or large parameters present) are scaled in order to be nominally of order unity. Thus, here $\hat{M}\hat{g}$ is the scaled weight $\hat{W}$ of the body. It is assumed that the typical *y* scale in the gap underneath the body is also of order *δ*, small compared with the *O*(1) length scale of *x*, but still large compared with the representative viscous thickness at large Reynolds numbers *Re* (that thickness being typically of order *Re*^−1/2^ in the laminar regime). In consequence, the flow itself is described formally by the classical boundary layer equations without a viscous contribution, yielding the so-called thin layer or, in another context, the shallow-water system for an effectively inviscid fluid. It is assumed in addition that the body, moving in response to the forces from the fluid motion, does so over time scales that are comparable with the time scales of the fluid motion and thereby has an appreciable effect on the fluid flow. The present assumptions imply that the flow over the horizontal length scale of order unity remains irrotational to leading order almost everywhere (since vorticity is conserved along particle paths) and so the scaled vorticity is zero. Thin-layer dynamics in which the vorticity is dominated by its ∂*u*/∂*y* component therefore require that *u* = *u*(*x*, *t*) does not depend on *y*, which forces *v* through continuity to change in *y* from zero at zero *y* to a value consistent with the kinematic condition at the unknown position of the moving lower surface of the thin body. Thus, the governing equations are

2.1*a* $$\begin{array}{r} \frac{\partial H}{\partial t}+\frac{\partial }{\partial x}(uH)=0, \end{array}$$

2.1*b* $$\begin{array}{r} \frac{\partial u}{\partial t}+u\frac{\partial u}{\partial x}=-\frac{\partial p}{\partial x}, \end{array}$$

2.1*c* $$\begin{array}{r} \;\text{where}\;H(x,t)=-f_{u}(x)+h(t)+(x-\sigma )\;\theta (t). \end{array}$$

Here H(x,t) denotes the unknown scaled thickness of the thin gap depending on the lower surface shape of the body and its orientation defined in (2.1c) above; *f*_*u*_(*x*) is the prescribed shape of the underbody. The contributions *h*(*t*) and *θ*(*t*) are owing to changes in the lateral location and orientation of the body, respectively, and are prescribed at the beginning in (2.1d). We remark that the real orientation angle is small, being *θδ* where *θ* can take any finite value in principle. Considering (2.1a–*c*) further, the kinematic condition yields (2.1a) while (2.1b) is the dominant streamwise momentum balance, with *p* being dependent only on *x*, *t* by virtue of the normal momentum balance, as in boundary layer theory.

Concerning initial and boundary conditions, at the initial contact point *x* = *σ* the constraints (2.1d) below come from the requirement of zero minimum gap width for the smooth shapes considered herein. Also, the condition (2.1e) below allows for a jump across the leading-edge Euler zone at *x* = 0 + . Since there is a quasi-steady local Euler flow around the leading edge [12,24], the quasi-steady Bernoulli condition (2.1e) is valid in the present unsteady flow scenario. The reason for the (Kutta) requirement (2.1f) below at the trailing edge of the body is that on top of the body the pressure varies typically by only a small amount of order *δ* throughout the external flow compared with its characteristic *O*(1) variation within the gap. Physically, the leading-edge jumps are induced by the necessity of the equi-pressure condition at the trailing edge (see [12,24]). The flow behind the body has no effect here to leading order. In consequence, we have the conditions

2.1*d* $$\begin{array}{r} h(0)=f_{u}(\sigma );\;\theta (0)=f_{u}^{\prime }(\sigma ); \end{array}$$

2.1*e* $$\begin{array}{r} p+\frac{1}{2}u^{2}=\frac{1}{2}\;\text{at}\;x=0+, \end{array}$$

2.1*f* $$\begin{array}{r} \;\text{and}\;p=0\;\text{at}\;x=1. \end{array}$$

These conditions are coupled with the body-motion equations in (2.1g,*h*). The unknowns $C_{L}(t),C_{M}(t)$ are, respectively, the scaled evolving lift and moment coefficients. The dimensional mass is $\rho^{*}{l^{*}}^{2}\hat{M}/\delta$, while the dimensional moment of inertia is $\rho^{*}{l^{*}}^{4}\hat{I}/\delta$. Also the acceleration due to gravity is $\delta {u^{*}}^{2}\hat{g}/l^{*}$ in dimensional terms. The Froude number is ${\left(\delta \hat{g}\right)}^{-1}$, whereas the Richardson number is $\delta \hat{g}$. Here $\hat{I}<\hat{M}/4$ from its definition. Thus, the equations for the body motion are

2.1*g* $$\hat{M}\frac{d^{2}h}{dt^{2}}=C_{L},\;\text{with}\;C_{L}=\int_{0}^{1}p(x,t)\;dx-\hat{W};$$

and

2.1*h* $$\hat{I}\frac{d^{2}\theta }{dt^{2}}=C_{M},\;\text{with}\;C_{M}=\int_{0}^{1}\left(x-\sigma )p(x,t\right)\;dx.$$

The task in general is to solve the nonlinear system (2.1a)–(2.1h) for *h*, *θ*, *u*, *p* and this is addressed successively for early times *t*, *O*(1) times and late times in the following sections.

## 3. Early behaviour

**Figure 2.**
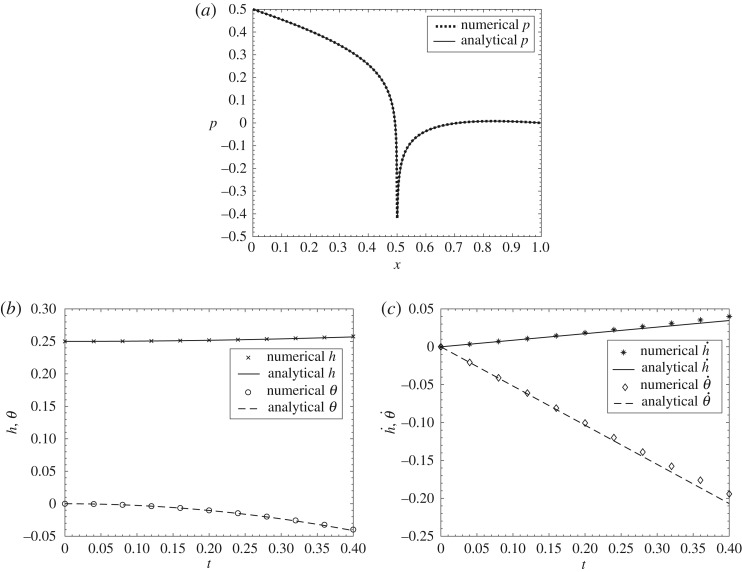
Comparison between small-time analytical solutions and numerical solutions for $\hat{M}=0.5$, $\hat{g}=0.24$ at early times (*a*) pressure at time *t* = 0 + , (*b*) *h* and *θ* against *t*, (*c*) $\dot{h}$ and $\dot{\theta }$ against *t*.

An investigation of the behaviour of the system at small times *t* proves to provide insight. This not only leads on to a comparison with direct numerical work (in figure 2 and in appendix A) but also yields a lift-off criterion.

The body is assumed to be initially at rest on the surface when the fluid flow is begun impulsively at time *t* = 0. Two flow regions are present at small positive *t*: one is affected by the complete underbody shape for *x* of order unity, specifically for 0 < *x* < *σ*, *σ* < *x* < 1, and the other closely surrounds the original contact point where the local body shape, being smooth, is *O*((*x* − *σ*)^2^) and is comparable with the initially small gap width which is *O*(*t*^2^) because the initial body acceleration is expected to be uniform. The reason for the uniformity rests in an argument based on orders of magnitude. In the two spatial dimensions present here, the boundary conditions in (2.1e,*f*) suggest that generally the pressure *p* should be of order unity at most and that implies pressure force contributions of order unity on the right-hand sides of the body movement balances in (2.1g,*h*). The left-hand sides then indicate that body-acceleration responses (d^2^*h*/d*t*^2^, d^2^*θ*/d*t*^2^) of order unity are likewise to be expected in general. Detailed working subsequently shows that *u* is of order *t* in the current setting in order to balance the pressure gradient in (2.1b) against the acceleration term ∂*u*/∂*t* and this confirms the *O*(1) pressure estimate above. We now investigate the fluid–body interaction as the lifting starts and in particular clarify the contributions from the inner and outer regions, which in one respect turn out to have similar degrees of importance. The main details are described in sections (a)–(c). In physical terms, the outer region in (a) suffers only small perturbations in height and inclination of the body compared with the initial state but these turn out to be sufficient to cause significant pressure forces to act over the majority of the underbody. The small inner region in (b) by contrast has height and inclination effects of order unity relative to the initial state as anticipated earlier in this paragraph and the pressure response is found to be logarithmically large but it acts only over the small length scale where *x* − *σ* is of order *t* in region (b) and so contributes only a comparatively minor influence on the lift and moment exerted on the body early on. The matching between the two regions is presented in (c) together with the resulting predictions for the changes in height and inclination of the body at small times.

### (a) Outer region

In view of the initial state and the uniform body accelerations d^2^*h*/d*t*^2^, d^2^*θ*/d*t*^2^ anticipated in the previous paragraph, we should expect the small-time expansions of the height *h* and inclination *θ* to contain contributions of order unity (from the initial fluid-filled gap) and *t*^2^ (from acceleration). For most *x* of *O*(1), the gap width, velocity and pressure thus develop at small times according to

3.1 $$(H,u,p)=(H_{0}(x),0,p_{0}(x))+(t^{2}H_{1}(x),tu_{1}(x),tp_{1}(x))+\cdots ,$$

with the dominant term of the gap width being defined by

3.2 $$H_{0}(x)=-f_{u}(x)+h_{0}+(x-\sigma )\theta_{0},$$

where the constants are *h*_0_ = *h*(0), *θ*_0_ = *θ*(0) in (2.1d). The pressure variation is *O*(1) because of the end constraints (2.1e,*f*) as mentioned before, so the body-motion balances (2.1g,*h*) are in agreement with *h*, *θ* variations of *O*(*t*^2^) such that

$$h=h_{0}+h_{2}t^{2}+\cdots ,\;\theta =\theta_{0}+\theta_{2}t^{2}+\cdots ,$$

while the *tu*_1_ term is inferred from the balance in (2.1a). Here the unknown constants *h*_2_, *θ*_2_ are proportional to the body acceleration coefficients. Substituting (3.1) into (2.1a) and integrating in *x* leads to the velocity in the outer region, of the form

3.3 $$u_{1}(x)=\frac{1}{H_{0}(x)}\{-2h_{2}(x-\sigma )-\theta_{2}{\left(x-\sigma \right)}^{2}+c_{1}\},$$

where *c*_1_ is the integration constant. Next, integrating the momentum equation (2.1b) with respect to *x* at leading order and considering (2.1e,*f*) yields the leading term of the pressure as

3.4 $$p_{0}(x)=\left\{ \begin{array}{ll} -\int_{0}^{x}u_{1}(\hat{x})\;d\hat{x}+\frac{1}{2}, & x\in [0,\sigma ),\\ -\int_{1}^{x}u_{1}(\hat{x})\;d\hat{x}, & x\in (\sigma ,1]. \end{array}\right.$$

Matching below implies that *c*_1_ is zero, leaving negligible inertial effects here. Also we observe the singular behaviour

3.5 $$p_{0}∼\frac{2h_{2}}{B}\ln|x-\sigma |+\pi_{\pm }\;\text{as}\;x\to \sigma_{\pm },\;\text{where}\;\pi_{+}=-\int_{1}^{\sigma }u_{1}\;dx,\;\pi_{-}=\frac{1}{2}-\int_{0}^{\sigma }u_{1}\;dx,$$

and the known constant $B=f_{u}^{\prime \prime }/2$ from the expression for the gap width near the original contact point. Next, the inner region near the initial contact *x* = *σ* needs to be studied.

### (b) Inner region

In the inner region a nonlinear effect asserts itself in terms of the gap width, with the solution taking the form

3.6 $$(H,u,p)=(t^{2}H_{2}^{*}(\xi ),\;u_{0}^{*}(\xi ),\;\frac{2h_{2}}{B}\ln(t)+p_{0}^{*}(\xi ))+\cdots ,$$

for *x* near the lift-off point: *x* = *σ* + *tξ* with *ξ*∼*O*(1). The scalings stem directly from those in the outer region. From substitution into (2.1a–*d*) and matching, the leading contributions satisfy

3.7*a* $$\begin{array}{r} 2H_{2}^{*}-\xi H_{2}^{*\prime }+{\left(u_{0}^{*}H_{2}^{*}\right)}^{\prime }=0, \end{array}$$

3.7*b* $$\begin{array}{r} -\xi u_{0}^{*\prime }+u_{0}^{*}u_{0}^{*\prime }=-p_{0}^{*\prime }, \end{array}$$

3.7*c* $$\begin{array}{rl} \text{where}\; & H_{2}^{*}(\xi )=B\xi^{2}+h_{2}, \end{array}$$

3.7*d* $$\begin{array}{r} \;\text{and}\;\{u_{0}^{*},p_{0}^{*}\}∼\{\alpha \xi^{-1},-\alpha \ln(\xi )+O(1)\}\;\text{as}\;\xi \to \pm \infty . \end{array}$$

The prime (′) denotes an ordinary derivative with respect to *ξ*. Here the constant $\alpha =-2h_{2}/B$ in (3.7d) for matching. The leading order terms in the local velocity and pressure expansion from (3.7a–*c*) are therefore as follows,

3.8 $$u_{0}^{*}(\xi )=\frac{-2\xi h_{2}-c_{0}^{*}}{B\xi^{2}+h_{2}},$$

and

3.9 $$p_{0}^{*}(\xi )=p_{0}^{*}(0)+\xi u_{0}^{*}(\xi )-\int_{0}^{\xi }u_{0}^{*}\;d\xi -\frac{1}{2}{u_{0}^{*}(\xi )}^{2}+\frac{1}{2}{u_{0}^{*}}^{2}(0),$$

where c*_0_, p*_0_(0) are integration constants to be determined.

### (c) Matching, and body motion

Matching the velocities and pressures in the inner and outer regions yields *c*_1_ = 0 in (3.3) and the constant c*_0_ is determined by

3.10 $$\frac{c_{0}^{*}\pi }{{\left(h_{2}B\right)}^{1/2}}=-\int_{1}^{\sigma }u_{1}\;dx-\frac{1}{2}+\int_{0}^{\sigma }u_{1}\;dx.$$

Similarly, p*_0_(0) can be found, and this completes the *u*, *p* solutions. Only the outer region controls the main body motion at leading order but, to clarify, the inner region completes the starting condition for numerical work as well as ensuring complete physical sense. Here the body movement relations (2.1g,*h*) yield the leading order contributions in the scaled height and inclination of the body at small times in the form

3.11*a* $$2\hat{M}h_{2}=\int_{0}^{\sigma }p_{0}(x,t)\;dx+\int_{\sigma }^{1}p_{0}(x,t)\;dx-\hat{W},$$

and

3.11*b* $$2\hat{I}\theta_{2}=\int_{0}^{\sigma }(x-\sigma )p_{0}(x,t)dx+\int_{\sigma }^{1}(x-\sigma )p_{0}(x,t)dx.$$

We need the right-hand side of (3.11a) to be positive in order that the body can lift off from the surface. Substitution of *p*_0_(*x*) from (3.4) into the system (3.11a,*b*) then leads to two linear equations

3.12*a* $$\begin{array}{rl} 2\hat{M}h_{2} & =\frac{\sigma }{2}+h_{2}I_{1}+\theta_{2}I_{2}-\hat{W}, \end{array}$$

3.12*b* $$\begin{array}{rl} 2\hat{I}\theta_{2} & =\frac{-\sigma^{2}}{4}+h_{2}I_{2}+\theta_{2}I_{3}, \end{array}$$

with constants

3.12*c* $$I_{1}=-2\int_{0}^{1}\frac{{\left(x-\sigma \right)}^{2}}{H_{0}(x)}\;dx,\;I_{2}=-\int_{0}^{1}\frac{{\left(x-\sigma \right)}^{3}}{H_{0}(x)}\;dx,\;I_{3}=-\frac{1}{2}\int_{0}^{1}\frac{{\left(x-\sigma \right)}^{4}}{H_{0}(x)}\;dx,$$

for the two unknowns *h*_2_, *θ*_2_. If the body is symmetric with *σ* = 1/2, then $I_{2}$ is identically zero.

Comparisons between the early-time predictions and the results of numerical simulations are presented in figure 2 for the particular case of scaled mass $\hat{M}$ equal to 0.5 and scaled gravity $\hat{g}$ equal to 0.24, with the scaled moment of inertia kept as 1/5 of the scaled mass. Further results and comparisons are shown in appendix A, where the numerical procedure is also described. The comparisons show quite close agreement at small times and tend to support both the direct numerical work and the asymptotic analysis.

In summary, the investigation of the early behaviour has shown a multi-structure occurring, with relatively large pressures and substantial flow velocities being induced very close to the original contact point with the ground. Elsewhere between the body and the ground substantial pressure variations are also provoked but with relatively minor flow velocities. The latter pressures act to drive the early movement of the body at the leading order. The next issue concerns, quantitatively, the question of whether lift-off actually occurs or not.

## 4. Lift-off criterion

The lift-off requirement is simply that *h*_2_ needs to be positive because of the nature of the gap width near its minimum value as displayed in (3.7c). If we consider a body having a general shape, using the relationship $\theta_{2}=(h_{2}I_{2}-\sigma^{2}/4)/\beta$ in (3.12b) where $\beta =(2\hat{I}-I_{3})$, (3.12a) becomes

4.1 $$\gamma h_{2}=\frac{\sigma }{2}-\hat{W}-\frac{I_{2}}{\beta }\frac{\sigma^{2}}{4},\;\text{with}\;\gamma =\left(\alpha -\frac{I_{2}^{2}}{\beta }\right),\alpha =(2\hat{M}-I_{1}).$$

We note that $I_{1}<0,I_{3}<0,\alpha >0,\beta >0$, while $I_{2}$ can either be negative or positive. If the scaled mass $\hat{M}$ and moment of inertia $\hat{I}$ are sufficiently large that *γ* > 0, then from (4.1) lift-off requires $\hat{M}\hat{g}<\sigma /2-\sigma^{2}/4\beta I_{2}$ by virtue of *h*_2_ > 0.

As a main example, for a parabolic-shaped body *f*_*u*_(*x*) = *κx*(1 − *x*) with curvature 2*κ* constant, for any *σ*, the criterion in (4.1) becomes

4.2 $$\hat{M}\hat{g}<\frac{\sigma }{2}+\frac{\sigma^{2}}{8}(1-2\sigma ){\left(2\hat{I}\kappa +\frac{1}{6}{\left(1-\sigma \right)}^{3}+\sigma^{3}\right)}^{-1}.$$

**Figure 3.**
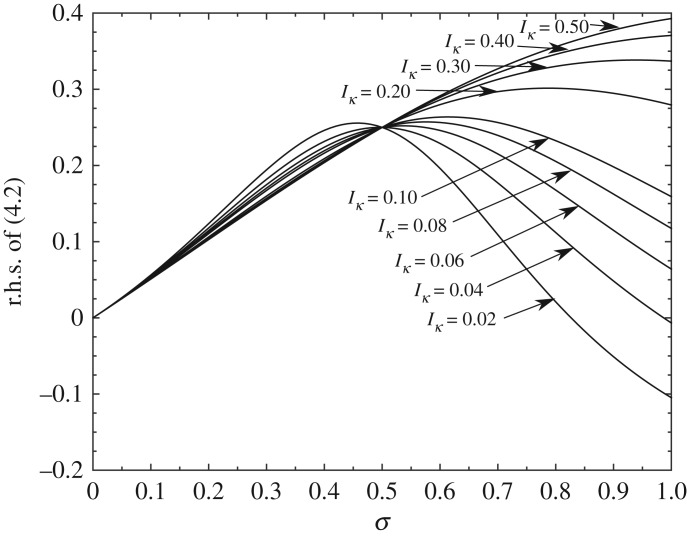
The right-hand side of (4.2) versus the original contact location *σ* for varying $\hat{I}\kappa$ (shown as *I*_*κ*_) ranging from 0.02 to 0.5. Body has a parabolic shape.

Figure 3 shows the right-hand side of (4.2) versus *σ* for a range of $\hat{I}\kappa$. At small *σ* values $\hat{M}\hat{g}$ has to be small for lift-off, whereas *σ* near unity allows lift-off for larger $\hat{M}\hat{g}$. In between there is a significant range of *σ* for which lift-off does not occur. Further, at *σ* = 1/2, the lift-off requirement is $\hat{M}\hat{g}<1/4$ in scaled terms, in keeping with (3.12a), i.e. the dimensional incident flow speed must exceed a critical value.(Other shapes lead to a more complex response.)

**Figure 4.**
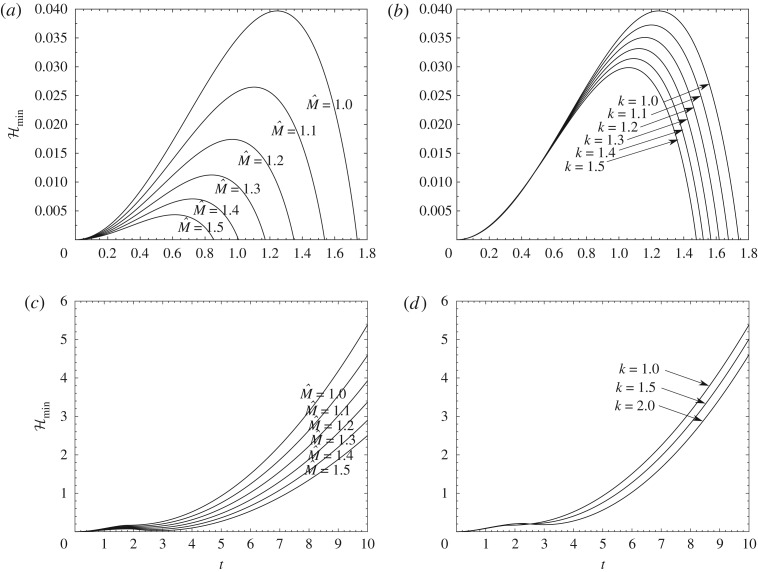
Numerical solutions of the minimum gap width $H_{\min}$ against *t* in which the body returns to the surface in (*a*, *b*) but flies away in (*c*, *d*). The effect of scaled mass $\hat{M}$ ranging from (*a*, *c*) 1 to 1.5. The effect of *κ* (written as *k*) ranging from (*b*) 1 to 1.5 and (*d*) 1 to 2. In (*a*, *c*), the scaled moment of inertia $\hat{I}$ also changes according to $\hat{I}=\Gamma \hat{M}$, for *Γ* = 0.2. Here a symmetrical sinusoidal shaped body is investigated; *f*_*u*_ = *κ*sin(*πx*).

**Figure 5.**
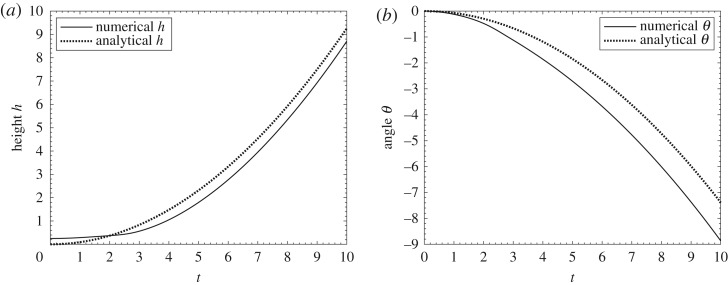
(*a*) Comparison between numerical and long-time analytical solutions for (*a*) scaled height *h*, (*b*) scaled angle *θ* for a fly-away case. Body has symmetric parabolic shape *f*_*u*_ = *x*(1 − *x*), *σ* = 0.5, scaled mass $\hat{M}=1$, moment of inertia $\hat{I}=0.2$, and $\hat{g}=0.1$.

Figures 4 and 5 show numerical evolutions of the system (2.1a–*h*). These numerical solutions were obtained by use of a finite-difference scheme similar to that in [24]. Appendix A describes the scheme used. The results in figure 4 are for cases where the body, having a sinusoidal shape, lifts off but, depending on the conditions, either returns to the ground after a finite time or flies away as time increases. This tends to confirm that different outcomes can occur for an underbody shape which is less simple than the parabolic shape. In the examples shown in figure 4*c*,*d*, each gradual dip of the minimum gap width with time indicates that a return to the ground almost occurs at some finite time, after which the trend towards a fly-away event takes control. Figure 5 is for the parabolic underbody shape where the given constant curvature allows comparison with the prediction (4.2) at early times, and similarly with the prediction (4.1) for any other shapes. In the former case, if the prediction (4.2) is not satisfied then the body cannot lift off. For the configurations in figure 5, however, the prediction is satisfied, the body lifts off and indeed the departure from the ground continues to large times and, similarly to the behaviour found in figure 4*c*,*d*, produces a fly-away phenomenon with *h*, *θ* becoming large then. To emphasize, *θ* here can take any positive, zero or negative value as it is a scaled angle of inclination, with the true angle being *θδ* in radians and *δ* being small. In some cases *θ* can keep increasing in value and *h* does not increase fast enough to prevent an impact of the body with the ground, producing a return to the surface as in the examples of figure 4*a*,*b*, whereas in the cases of figure 5 the scaled height *h* rises sufficiently rapidly that such impact is avoided. A comparison is also shown in figure 5 with large-time analysis which is brought forward from §6 to highlight the long-term trend here.

## 5. For large mass and moment of inertia

For large values of the scaled mass and moment of inertia $\hat{M},\hat{I}$, we expect the time scales of the body movement (2.1g,*h*) to dominate over those associated with the fluid motion in (2.1a,*b*). Hence, since the typical *h*, *p*, *θ* remain of *O*(1), $t={\hat{M}}^{1/2}T$ with *T* being of order unity. Also $\hat{g}∼O({\hat{M}}^{-1})$, keeping weight $\hat{W}$ of order unity, and we take $\hat{I}=\hat{M}\Gamma$ with the constant *Γ* being *O*(1). The flow time derivatives in (2.1a,*b*) become negligible which gives us simple velocity and pressure solutions, leading on substitution into (2.1g,*h*) to

5.1*a* $$\ddot{h}=\frac{1}{2}\int_{0}^{1}(1-{\left(h(T)+(1-\sigma )\theta (T)\right)}^{2}H{\left(x,T\right)}^{-2})\;dx-\hat{W},$$

and

5.1*b* $$\Gamma \ddot{\theta }=\frac{1}{2}\int_{0}^{1}\left(x-\sigma )(1-{\left(h(T)+(1-\sigma )\theta (T)\right)}^{2}H{\left(x,T\right)}^{-2}\right)\;dx.$$

This yields two coupled ordinary differential equations [24] for *h*, *θ* in the case of the parabolic underbody shape. More significantly, however, even in the case of an arbitrary shape (5.1a,*b*) themselves suggest a large-*t* response in which

5.2 $$h,\theta \;\text{grow like}\;t^{2}\;\text{at large times}.$$

This produces integrands of order unity in (5.1a,*b*) and also gives independence from the shape *f*_*u*_(*x*) since, from (2.1c), H is dominated by the contributions *h* + (*x* − *σ*)*θ* of order *t*^2^ for almost all *x* values. We pursue this below in the general case.

## 6. Large-time behaviour

This analysis applies for *t*≫1 and general values of the parameters $\hat{M},\hat{I},\hat{g}$. Guided by the idea in the previous section, we see that the response at large times is that *h*, *θ* are of *O*(*t*^2^) as *p* typically must be *O*(1) by virtue of (2.1e). The resulting asymptotic description takes the form

6.1 $$(H,h,\theta ,u,p)=(t^{2}H_{2}(x),t^{2}h_{2},t^{2}\theta_{2},u_{0}(x),p_{0}(x))+\cdots .$$

Hence in (2.1a,*b*), the time derivatives $H_{t},u_{t}$ are negligible and simple quasi-steady relations hold again. The body shape contribution *f*_*u*_(*x*) likewise becomes negligible compared with the *h*, *θ* contributions. Also the Kutta condition (2.1f) leads to *u*_0_(1) = 1. The leading-order velocity *u*_0_ is therefore

6.2 $$u_{0}(x)=(h_{2}+(1-\sigma )\theta_{2}){\left(h_{2}+(x-\sigma )\theta_{2}\right)}^{-1}.$$

Substituting (6.2) into (2.1e) then gives the pressure

6.3 $$p_{0}(x)=\frac{1}{2}(1-{\left(h_{2}+(1-\sigma )\theta_{2}\right)}^{2}{\left(h_{2}+(x-\sigma )\theta_{2}\right)}^{-2}).$$

Hence the body-balance equations in (2.1g,*h*) yield

6.4*a* $$2\hat{M}h_{2}=-\frac{\theta_{2}}{2}{\left(h_{2}-\sigma \theta_{2}\right)}^{-1}-\hat{W},$$

and

6.4*b* $$4\Gamma \hat{M}\theta_{2}=\left(\frac{1}{2}-\sigma \right)+\frac{h_{2}}{\theta_{2}}\left(\frac{h_{2}+(1-\sigma )\theta_{2}}{h_{2}-\sigma \theta_{2}}\right)-\frac{1}{\theta_{2}^{2}}{\left(h_{2}+(1-\sigma )\theta_{2}\right)}^{2}\ln|\frac{h_{2}+(1-\sigma )\theta_{2}}{h_{2}-\sigma \theta_{2}}|.$$

With the solutions *h*_2_, *θ*_2_ thus determined, the leading-order terms in gap width, velocity and pressure solutions can now be determined.

Figure 5 shows a sample comparison between the numerical and the analytical solutions for the height *h* and angle *θ*. The analytical ones use the large-time asymptotic expansions in (6.4a,*b*). A close match is seen for both *h*, *θ*, with the near-constant differences between the numerical and the large-time asymptotic results being attributable to effects of higher order in the latter approach including an arbitrary constant shift in the origin of time. In this example, and others not shown here, the body lifts off and rotates but does not impact on the ground, instead continuing to depart from the ground.

**Figure 6.**
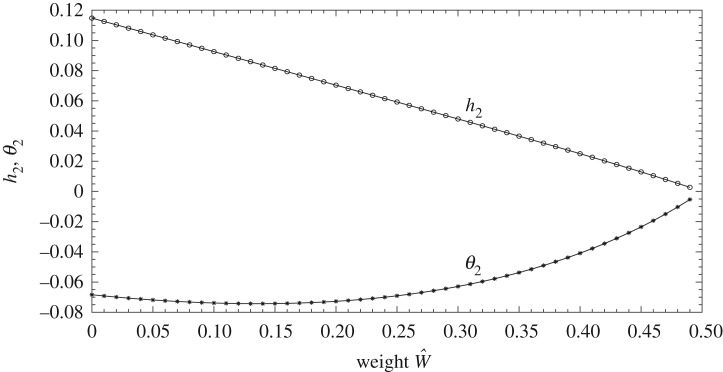
The coefficients *h*_2_, *θ*_2_ from (6.4a,*b*) in the large-time response versus scaled weight: $0<\hat{M}\hat{g}<0.5$ for $\sigma =0.5,\;\hat{M}=1$. This indicates the range for lift-off and fly-away for any body shape: see text. For scaled weights over the value 0.5 fly-away cannot occur.

Concerning the fly-away criterion, figure 6 plots the coefficients *h*_2_, *θ*_2_ versus scaled weight. A critical value $\hat{W}={\hat{W}}_{c}(=\frac{1}{2})$ emerges. We put

6.5 $$\hat{M}\hat{g}=\frac{1}{2}-\epsilon \;\text{with }\epsilon \text{ small and positive},$$

and then expand *h*_2_, *θ*_2_ in powers of *ϵ* such that $(h_{2},\theta_{2})=\epsilon ({\hat{h}}_{2},{\hat{\theta }}_{2})+\cdots$. It is found with $\hat{M}=1$, for example, that

6.6 $$(h_{2},\theta_{2})=\left(\frac{\epsilon }{2},-\epsilon \right)+\cdots .$$

The critical value 1/2 applies for any shape of the body.

In the critical case, the main balance of forces is between weight and the pressure force driven by the Bernoulli pressure head since *h*_2_, *θ*_2_ are small. The asymptotic predictions agree well with those in figure 6 as *t* increases. For a symmetric body for example, the (*h*_2_ + (1 − *σ*)*θ*_2_) contribution in (6.4b) implies that the trailing edge stops rising, whereas the leading edge height is still increasing.

## 7. Summary

The lift-off of a single solid body from a flat surface has been modelled, analysed and calculated and this leads to certain experimental links as discussed below. The critical value of scaled weight is found to be 1/4 for any symmetric body in order that the body can initially lift off from the surface. A lift-off criterion is also found for non-symmetric body shapes although the criterion in this case is more complex as it is very shape-dependent. The body can subsequently return to the surface for a range of scaled mass, moment of inertia, gravity and body shapes, but alternatively fly-away occurs. This depends to a large extent on a competition between the vertical force and the rotating force on the body at earlier times. The critical value of the scaled weight such that the body can fly away is found: the critical value 1/2 applies irrespective of the shape of body, which potentially may seem a powerful result although it is subject of course to the assumptions involved, including the thinness of the body and the lack of separation. Non-dimensionally the 1/4 factor at early times is due to Bernoulli pressure acting only on the front half of the underbody in contrast with the complete underbody at late times due to the body being far from the wall which yields the 1/2 factor.

If the leading edge of the body goes up that generally implies an increased lift whereas a descending leading edge is associated with downforce. Likewise, the body rotation produced can lead to an impact on the ground or continued departure from the ground. Here the ground effect reduces the possibility of lift-off by reducing the lift-off force due to a given fluid flow in comparison with the fly-away case. In dimensional terms for a body mass represented as ρ*_B_h*_B_l* where ρ*_B_ is the body density and h*_B_ is the mean body thickness the lift-off and fly-away criteria on the incident velocity can be written, respectively,

7a,b $$\frac{u^{*2}}{\left(h_{B}^{*}g^{*}\right)}>4\left(\frac{\rho_{B}^{*}}{\rho^{*}}\right),\;\frac{u^{*2}}{\left(h_{B}^{*}g^{*}\right)}>2\left(\frac{\rho_{B}^{*}}{\rho^{*}}\right),$$

(for a symmetric body in the case of (7.1*a*) with g* denoting gravity). We remark again that the overall results derived here are mostly illustrated by specific examples and that the parameter space is large. Nevertheless, (7.1*a*,*b*) appear to represent quite general criteria. Beyond a few significant experiments and observations (see in next paragraph), there tend to be limited comparisons possible with experiments and direct simulations owing to the different parameter ranges involved as well as the model assumptions.

Two particular points stand out in regard to experimental links and observations. The first is that the criteria above are on effective Froude numbers and are akin to Shield's condition [25–27] in sediment processes but without shear stresses, given the present model has negligible viscous effects and corresponding friction forces are small compared with the form forces due to pressure. Our study incorporating ground effect shows an evolution towards or away from fly-away and determines a precise coefficient (4 or 2) rather than the order of magnitude estimate of Shield, although this is for thin bodies. The second particular point is that, in a quite different setting, the comparison and broad agreement concerning the scalings associated with dust movement on the planet Mars still hold for the present work as in [12] since the present post-lift-off result agrees exactly in terms of its orders of magnitude with that in the previous pre-lift-off work based on normal force.

A basic explanation of fly-away is also provided by the present study via the original governing equations (2.1a,*b*) where the $H_{t},u_{t}$ contributions diminish when *t*≫1. Hence the quasi-steady Bernoulli relation holds for *p*. So then the body-motion balance involving $\hat{M}\ddot{h}$ leads to the requirement $\hat{M}\hat{g}<1/2$ in scaled terms, which simply balances weight against lift. In addition the lift-off condition is different from the fly-away condition because of the ground effect, implying that symmetric bodies for instance which are subject to flow velocities between the two values in (7.1) satisfy the fly-away condition but are unable to lift off, while a body that lifts off can either fly away or return to the ground.

Among the various major assumptions are the body thinness (or the gap thinness in the case of a thicker body), the given quasi-inviscid fluid and the flow irrotationality over the scales present. The assumptions are made in a first-go broad approximation for the nonlinear fluid–body interaction arising in lift-off, return or fly-away. These seem to yield results relevant to some applications in addition to further understanding, subject to the comments and caution above.

Other interesting matters have still to be addressed. The effect of incident shear in the oncoming flow has not been considered yet in the present context and neither have three-dimensionality or viscous effects: see the latter in the recent analyses of [22,23] and shear effects in the recent experiments of [9]. We have supposed thin turbulent wall layers but we can certainly expect separation to take place in the laminar regime. The assumption of separation-free flow in the context of any laminar wall sublayers is certainly open to question on physical grounds if a more detailed model is to be developed and explored, given that sublayer separation may influence substantially the flow dynamics over a wide range of parameters and alter the conditions for lift-off of the body and return to the ground, if not the conditions for fly-away where the ground effect is reduced. The influence is dependent, however, on the body surface and ground conditions in detail, for example, whether the body is moving horizontally relative to the ground and whether the solid surfaces involved are rough or smooth. On a larger time scale, the normal pressure gradient within the fluid flow comes into play significantly as the ground effect diminishes. The incorporation of non-symmetric bodies as mentioned previously and likewise more complex bodies such as those with concavities offer interesting challenges for the future. Other possible extensions might be to reptation, clashes, body flexibility, and investigating the effects of the surface shape on which the body lies initially. The extension to several bodies is of further concern.

## Figures and Tables

**Figure 7.**
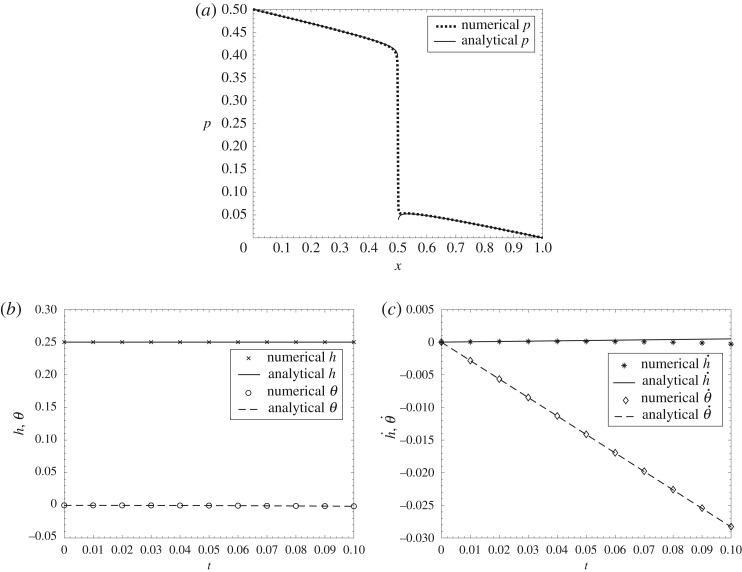
Comparison between small-time analytical solutions and numerical solutions for $\hat{M}=1$, $\hat{g}=0.24$ at early times (*a*) pressure at time *t* = 0 + , (*b*) *h* and *θ* against *t*, (*c*) $\dot{h}$ and $\dot{\theta }$ against *t*.

**Figure 8.**
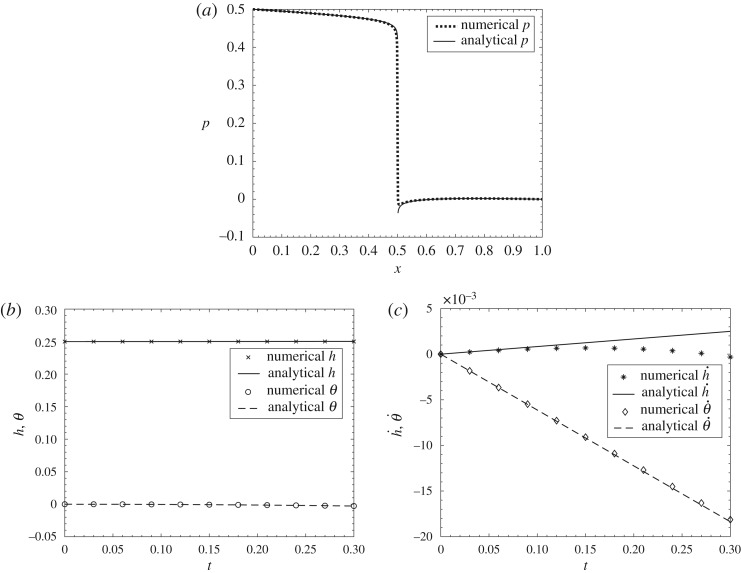
Comparison between small-time analytical solutions and numerical solutions for $\hat{M}=5$, $\hat{g}=0.04$ at early times (*a*) pressure at time *t* = 0 + , (*b*) *h* and *θ* against *t*, (*c*) $\dot{h}$ and $\dot{\theta }$ against *t*.

## Data accessibility

This article does not contain any additional data.

## Appendix A. Numerical study of the complete interaction

The numerical treatment uses finite differencing. A brief overview of the algorithm is as follows. First, we specify the initial conditions on body position and fluid flow velocity. Then the scheme advances the body to its new position based on (2.1g,*h*) and finds the velocity and pressure of the fluid using (2.1a–*f*). The scheme checks for the body returning to the surface and then either finds the body's new position, fluid velocity and pressure as above or stops when the body returns to the surface.

We expand on this description in a little more detail as follows. Initially, the body is placed horizontally on the surface. To proceed by a specified small time step *δt* (typically 5 × 10^−5^) to the next time step, the scaled trailing-edge pressure is forced to zero in the following manner. Having performed an integration in (2.1a), discretization yields

A 1 $$u_{i}[-f_{u}(x)+h+(x-\sigma )\theta ]=[c-\dot{h}(x-\sigma )-\frac{1}{2}{\left(x-\sigma \right)}^{2}\dot{\theta }]$$

which is solved for *u* = *u*_*i*_ values at the next time step from *x* = 0 to *x* = 1 (*i* = 1 to *i* = *N*) as we are only interested in this region where the body is located. The constant *c* is to be found. The spatial steps *δx* are taken typically as 0.01 (*N* = 101 steps) or smaller. The *c* value is found directly using the trailing-edge pressure condition. Next, (2.1b) is discretized using:

A 2 $$p_{i}^{j}=\frac{1}{2}(1-{\left(u_{i}^{j}\right)}^{2})-\frac{\delta x}{\delta t}\sum_{k=1}^{i}(u_{i}^{j}-u_{i}^{j-1})\;\text{for}\;i=2,\ldots ,N,\;\text{and}\;j=1,\ldots ,K.$$

Here the subscript and superscript denote a variable's spatial and time indexing, respectively, while *K* is the total number of time steps. The pressure values *p*_*i*_ are therefore determined via (A 2). The trailing-edge pressure constraint is then addressed using a secant method. Next, the starting value *u*_1_ is updated by carrying out iterations and continuing these until the difference between the required value of the trailing-edge pressure and *p*_*N*_ is sufficiently small. Here all the terms d*h*/d*t*, *h*, d*θ*/d*t*, *θ* are renewed by integrating (2.1g,*h*) with an implicit Euler method and using the trailing-edge pressure constraint *p*_*N*_ = 0. To advance to the next time step, the same procedure is performed with the *h*^*j*−1^ being updated to *h*^*j*^, *θ*^*j*−1^ to *θ*^*j*^, *p*^*j*−1^_*i*_ to *p*^*j*^_*i*_, *u*^*j*−1^_*i*_ to *u*^*j*^_*i*_ and so on. The procedure is performed until the body either returns to the surface or in effect flies away. A range of scaled mass and moment of inertia values $\left(\hat{M},\hat{I}\right)$ are accommodated in the treatment as well as the effect of the scaled acceleration due to gravity $\hat{g}$.

Checks on the influences of the spatial and temporal step sizes are presented in detail in [24]. The sets of finite-difference solutions there are found to be robust as the step sizes are refined gradually.

Results and comparisons between the finite-difference solutions and the small-time analysis are shown in figures 7 and 8 (in addition to the solutions given in figures 2–6). The results in figures 7 and 8 enlarge the parameter range presented here, and again, as in figure 2, the agreement between the numerical and analytical findings seems encouragingly close.
